# Endonuclease Restriction-Mediated Real-Time Polymerase Chain Reaction: A Novel Technique for Rapid, Sensitive and Quantitative Detection of Nucleic-Acid Sequence

**DOI:** 10.3389/fmicb.2016.01104

**Published:** 2016-07-13

**Authors:** Yi Wang, Yan Wang, Lu Zhang, Machao Li, Lijuan Luo, Dongxin Liu, Hua Li, Xiaolong Cao, Shoukui Hu, Dong Jin, Jianguo Xu, Changyun Ye

**Affiliations:** ^1^State Key Laboratory of Infectious Disease Prevention and Control, Collaborative Innovation Center for Diagnosis and Treatment of Infectious Diseases, National Institute for Communicable Disease Control and Prevention, Chinese Center for Disease Control and PreventionBeijing, China; ^2^Pathogenic Biology Institute, University of South ChinaHengyang, China; ^3^Department of Microbiology, Guiyang Medical UniversityGuiyang, China

**Keywords:** ET-PCR, real time PCR, PCR, LoD, nucleic acid detection

## Abstract

The article reported a novel methodology for real-time PCR analysis of nucleic acids, termed endonuclease restriction-mediated real-time polymerase chain reaction (ET-PCR). Just like PCR, ET-PCR only required one pair of primers. A short sequence, which was recognized by restriction enzyme BstUI, was attached to the 5′ end of the forward (F) or reverse (R) PCR primer, and the new F or R primer was named EF or ER. EF/ER was labeled at the 5′ end with a reporter dye and in the middle with a quenching dye. BstUI cleaves the newly synthesized double-stranded terminal sequences (5′ end recognition sequences and their complementary sequences) during the extension phase, which separates the reporter molecule from the quenching dye, leading to a gain of fluorescence signal. This process is repeated in each amplification cycle and unaffected the exponential synthesis of the PCR amplification. ET-PCR allowed real-time analysis of single or multiple targets in a single vessel, and provided the reproducible quantitation of nucleic acids. The analytical sensitivity and specificity of ET-PCR were successfully evaluated, detecting down to 250 fg of genomic DNA per tube of target pathogen DNA examined, and the positive results were generated in a relatively short period. Moreover, the practical application of ET-PCR for simultaneous detection of multiple target pathogens was also demonstrated in artificially contaminated blood samples. In conclusion, due to the technique’s simplicity of design, reproducible data and low contamination risk, ET-PCR assay is an appealing alternative to conventional approaches currently used for real-time nucleic acid analysis.

## Introduction

The invention of polymerase chain reaction (PCR) revolutionized the field of biological research and molecular diagnostics, since specific target sequence in genome can be amplified to detectable levels within a few hours (Moore, 2005; Wang et al., 2015a). In order to achieve amplification, the PCR technology employed a cyclic three key steps: initiation, annealing and elongation, all of which interplay with a decreasing or increasing temperature (Mullis and Faloona, 1987). The PCR amplification products were detected by electrophoresis on agarose gels with ethidium bromide staining (Law et al., 2015; Wang et al., 2015b, 2016). However, the post detection procedure was time-consuming, required additional equipment and reagents, and did not allow real-time detection, posing an obstacle for rapid analysis of nucleic acid sequences.

In order to overcome PCR limitations, real-time PCR technology, which has been devised in many molecular biology and clinical laboratories for rapid analysis applications, effectively alleviated the use of a post-PCR analysis and became an appealing alternative to the conventional PCR (Arya et al., 2005; Ettenauer et al., 2014). The real-time PCR can monitor the PCR products formation continuously in the entire amplification by measuring the fluorescent signal yielded by intercalating dyes or specific dual-labeled probes (Kubista et al., 2006). In the last 15 years, many fluorescent systems have been established for real-time PCR, and the most commonly used real-time PCR chemistries were double-stranded DNA (dsDNA) intercalating molecules (such as SYBR green) and fluorophore-labeled oligonucleotides (such as primer-based probes and double-dye probes; Oppliger et al., 2011). The dsDNA-binding fluorescent dye, which could bind any dsDNA, displayed several disadvantages for nucleic acid sequence analysis, for example detection of non-specific amplification products, high tendency to inhibit PCR at higher concentration and preferential binding to specific DNA sequences (Gašparič et al., 2010). The use of primer-based probes (such as hairpin primer-probes) could generate amplification of unspecific products or dimer-primers during the PCR reaction (Navarro et al., 2015). The technologies involving double-labeled probes, such as TaqMan and molecular beacon probes, could complicate design of the fluorescent oligonucleotide probe for real-time measurement of specific amplified products (Arya et al., 2005). Herein, an alternative technique, which can overcome the technical difficulties posed by current real-time PCR assays, is in continuous demand.

In this report, a novel mode of real-time PCR, termed endonuclease restriction-mediated real-time polymerase chain reaction (ET-PCR), was devised, which achieved the analysis of nucleic acid sequences with easy design, low contamination risk, high reproducibility, sensitivity and specificity. The ET-PCR system merges endonuclease restriction digestion and real-time fluorescence detection with PCR chemistry, which can simultaneously detect and differentiate multiple targets in a single reaction in a relatively short period. Here, we expound the basic ET-PCR principle and demonstrate an application of the novel methodology.

## Materials and Methods

### Design of ET-PCR Primers

**FIGURE 1:**
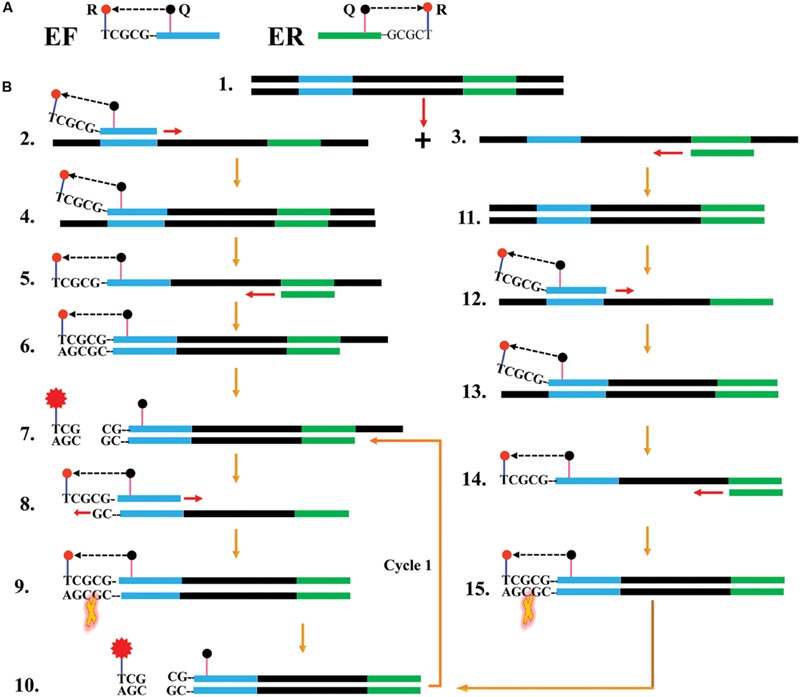
**Reaction mechanism of endonuclease restriction-mediated real-time polymerase chain reaction (ET-PCR). (A)** The schematic depiction of a new forward/reverse primer (EF/ER). EF/ER primer, which is an extension of the conventional forward (F)/reverse (R) primer with a short sequence (Ss, 5′-TCGCG-3′, its complementary sequence termed Cs) at the 5′ end, contained a reporter dye (R) at the 5′ end and a quencher dye (Q) in the middle. Due to their close proximity, the fluorescence emitted from the reporter molecules was quenched by the quenching dye. **(B)** Outline of ET-PCR chemistry, with EF and R primers. For clarity, the ER primer was not listed. Firstly, the double-stranded DNA was denatured into single-stranded DNA at high temperature, and the two single-stranded synthetic primers (EF and R) annealed to the target DNA strands (Steps 1–3). The EF primer was extended and the newly synthesized product entered a cyclic two steps process, thus a target double-trended terminal sequence was formed (Steps 4–6). BstUI cleaves the sequence 5′-TCG-3′ and its Cs, and the reporter molecule was separated from the quenching molecule leading to a gain of fluorescent signal (Step 7). The double-trended DNA (Step 7) was used as templates for further RT-PCR amplification, resulting gain of exponential fluorescence signals (Cycle1, Steps 8–11). For the R primer, the reaction process was similar to EF primer, and the fluorescence signals also were released in the amplification process (Steps 11–15). The ET-PCR process was repeated in each cycle without influencing with exponential synthesis of the PCR products.

**FIGURE 2:**
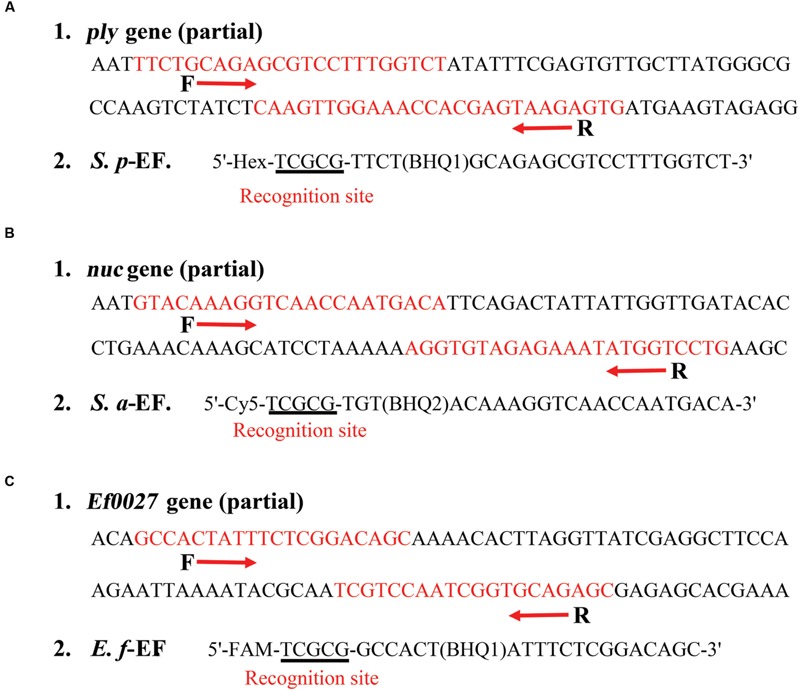
**The scheme lists the design and sequences of three sets of ET-PCR primers. (A1)**, **(B1)**, and **(C1)**: The nucleotide sequences of the sense strand of *ply* gene (part), *nuc* gene (part) and *Ef0027* gene (part) are shown. The locations of the primer sites are marked in red, right and left arrows indicate the sense and complementary sequences that are used. **(A2)**, **(B2)**, and **(C2)**: The modifications and sequences of three EF primers. The restriction enzyme recognizing sites are underlined.

Based on the *ply* gene (GenBank accession EF368014) of *Streptococcus pneumoniae* (*S. pneumoniae*), *nuc* gene (GenBank accession EF529597) of *Staphylococcus aureus* (*S. aureus*) and *Ef0027* gene (GenBank accession 1198935) of *Enterococcus faecalis* (*E. faecalis*), three sets of ET-PCR primers were designed by primer software PRIMER PREMIER 5.0 according to the mechanism of ET-PCR (**Figure 1**; Corless et al., 2001; Liu et al., 2005; Yang et al., 2011). In order to achieve the simultaneous amplification in the same conditions and multiplex reactions, three sets of ET-PCR primers were designed to have similar physical characteristics. The melting temperatures of three sets of primers were between 58.8 and 63.2°C, the lengths of the primers between 20 and 26 bp, and the amplicon sizes ranged between 80 and 93 bp. Blast analysis validated that three sets of primers were specific for *S. pneumoniae, S. aureus*, and *E. faecalis*. The reporter dyes used were HEX, Cy5, and FAM, which could be analyzed in real-time system (Rotor-Gene Q, Qiagen) that was selected for conducting the ET-PCR reactions. The quenching dyes used were Black Hole Quencher-1 and Black Hole Quencher-2, which could prevent the emitted fluorescence of the reporter dyes. The design, locations and sequences of ET-PCR primers were displayed in **Figure 2** and **Table 1**. All of the oligomers were synthesized and purified by Tian-Yi Biotech (Beijing, China).

**Table 1:** Primers used in this study.

Primer name^a^	Sequences and modifications	Length	Genes
S.p-F	5′-TTCTGCAGAGCGTCCTTTGGTCT-3′	23 nt	*ply*
S.p-R	5′-CACTCTTACTCGTGGTTTCCAACTTG-3′	26 nt	
S.p-EF	5′-Hex-TCGCG-TTCT(BHQ1)GCAGAGCGTCCTTTGGTCT-3′	28 mer	
S.a-F	5′-TGTACAAAGGTCAACCAATGACA-3′	23 nt	*nuc*
S.a-R	5′-CAGGACCATATTTCTCTACACCT-3′	23 nt	
S.a-EF	5′-Cy5-TCGCG-TGT(BHQ2)ACAAAGGTCAACCAATGACA-3′	28 mer	
E.f-F	5′-GCCACTATTTCTCGGACAGC-3′	20 nt	*Ef0027*
E.f-R	5′-GCTCTGCACCGATTGGACGA-3′	20 nt	
E.f-EF	5′-FAM-TCGCG-GCCACT(BHQ1)ATTTCTCGGACAGC-3′	25 mer	

*^a^F, forward; R, reverse; EF, the new Forward; S. p, *S. pneumonia*; S. a, *S. aureus*; E. f, *E. faecalis*.*

### Bacterial Strains and DNA Extraction

Of the total of 113 stock strains used in this study were shown in **Table 2**. These strains were stored in 10% (w/v) glycerol broth at -70°C, and refreshed three times on blood agar at 37°C. Then, all strains were applied to enrich and extract genomic DNA templates according to the manufacturer’s instructions (QIAamp DNA minikits; Qiagen, Hilden, Germany). The extracted genomic templates were determined with ultraviolet spectrophotometer (NanoDrop ND-100, QingDaShiKe, Beijing, China) at A260/280 and stored at -20°C before they were used.

**Table 2:** Bacterial strains used in this study.

Bacteria	Serovar/species	Strain number (source of strain)^a^	Number of strains
*Streptococcus pneumoniae*	19A	ATCC700674	1
	14	ATCC6314	1
	19F	ATCC49619	1
	35B	ATCCBAA660	1
	23F	ATCC700669	1
	6A	ATCCBAA659	1
	6B	ATCCBAA658	1
	1	NCTC7465	1
	5	ICDC-SP112007	1
	23A	ICDC-SP028	1
	6C	ICDC-SP0261	1
	3	ICDC-SP31003	1
	18C	ICDC-SPM241	1
	9V	ICDC-SP0269	1
	9N	ICDC-SP0282	1
	15B	ICDC-SP0277	1
	U	Isolated strains (ICDC)	28
*Staphylococcus aureus*	U	ICDC-NPSau001	1
	U	ICDC-NPSau002	1
	U	ICDC-NPSau003	1
	U	ICDC-NPSau004	1
	U	ICDC-NPSau005	1
	U	Isolated strains (ICDC)	20
*Enterococcus faecalis*	U	ATCC51299	1
	U	ATCC35667	1
	U	Isolated strains (ICDC)	20
*Enterotoxigenic Escherichia coli*	U	Isolated strains (ICDC)	1
*Enteroaggregative E. coli*	U	Isolated strains (ICDC)	1
*Enteroaggregative E. coli*	U	Isolated strains (ICDC)	1
*Enteroinvasive E. coli*	U	Isolated strains (ICDC)	1
*Enterohemorrhagic E. coli*	U	EDL933	1
*Plesiomonas shigelloides*	U	ATCC51903	1
*Enterobacter cloacae*	U	Isolated strains (ICDC)	1
*Enteropathogenic E. coli*	U	ATCC35667	1
*Yersinia enterocolitica*	U	ATCC23715	1
*Bntorobater sakazakii*	U	Isolated strains (ICDC)	1
*Vibrio cholerae*	U	Isolated strains (ICDC)	1
*Shigella boydii*	U	Isolated strains (ICDC)	1
*Shigella flexneri*	1d	ICDCNPS001	1
*Vibrio parahaemolyticus*	U	Isolated strains (ICDC)	1
*Enterotoxigenic faecium*	U	ATCCBAA340	1
*Listeria monocytogenes*	4a	ATCC19114	1
*Vibrio vulnificus*	U	Isolated strains (ICDC)	1
*Samonella*	U	Isolated strains (ICDC)	1
*Campylobacter jejuni*	U	ATCC33291	1
*Pseudomonas aeruginosa*	U	Isolated strains (ICDC)	1
*Bacillus cereus*	U	Isolated strains (ICDC)	1

*^a^U, unidentified serotype; ATCC, American Type Culture Collection; NCTC, National Collection of Type Cultures; ICDC, National Institute for Communicable Disease Control Disease Control and Prevention, Chinese Center for Disease Control and Prevention.*

### The Standard ET-PCR Reaction

Amplification reaction mixtures of ET-PCR were carried out in a 25 μl reaction volume containing 0.4 μM EF, 0.4 μM R primer, 12.5 μl 2 × Premix (Takara Bio, Inc., Otsu, Japan) Ex Taq TM, 1.5 μl (15 U) of BstUI endonuclease, and 1 μl of DNA template. The assays were conducted using the PCR settings of pre-denaturation at 95°C for 60 s, 40 cycles of denaturation at 95°C for 5 s, and extension at 60°C for 40 s in real-time system (Rotor-Gene Q, Qiagen). Fluorescence readings were obtained using HEX, Cy5, and FAM channels. In order to further confirm the correct amplification of ET-PCR, additional strategy was chosen for analyzing the amplification products. The reaction products were analyzed by electrophoresis on 2% agarose gels with ethidium bromide staining. The reaction mixtures without genomic templates were used as a negative control.

### Evaluation of ET-PCR Assay Sensitivity

In order to test the analytical sensitivity of ET-PCR assay, ET-PCR were performed using serial dilutions (2.5 × 10^7^, 2.5 × 10^6^, 2.5 × 10^5^, 2.5 × 10^4^, 2.5 × 10^3^, 2.5 × 10^2^, 2.5 × 10^1^, and 2.5 × 10^0^ fg per microliter) of pure *S. pneumonia* (ATCC 700674), *S. aureus* (ICDC-NP001) and *E. faecalis* (ATCC 51299) genomic DNA templates. The genomic templates (1 μl) were added into the ET-PCR mixture and at least four replicates of each dilution were tested to define the limit of detection (LoD) of ET-PCR approach. Mixtures without DNA templates were selected as a negative control.

In this report, we compared the LoD of ET-PCR technique with qPCR (SYBR Green) and end-point PCR assays, and the serial dilutions of pure genomic DNA templates described above were applied to perform the qPCR and PCR amplifications. The qPCR reactions were performed in a final volume of 25 μl reaction volume containing 0.4 μM F, 0.4 μM R primer, 12.5 μl 2 × SYBR^®^ Premix Ex Taq^TM^ II (Takara Bio, Inc., Otsu, Japan) and 1 μl of DNA template. The qPCR reactions were conducted using the PCR settings of pre-denaturation at 95°C for 30 s, 40 cycles of denaturation at 95°C for 5 s, and extension at 60°C for 30 s in real-time system (Rotor-Gene Q, Qiagen). The reaction mixtures without genomic templates were used as a negative control.

End-point PCR reaction was carried out in a final volume of 20 μl containing 50 mM KCl, 10 mM Tris-HCl (pH 8.3), 1.5 mM MgCl_2_, 0.001% gelatin, 0.2 mM each of dNTPs, 0.4 μM each of F and R primers, 1 μl genomic templates, and 0.5 units of Taq DNA polymerase (ExTaq; Takara). The PCR programs contained the initial denaturation of 5 min at 95°C, followed by 32 cycles of 30 s at 95°C, 30 s at 58°C and 40 s at 72°C, and a final 5 min extension at 72°C in a LabCycler 96 Cycler (Senso Quest, Beijing, China). Amplicons were determined under UV fluorescence following electrophoresis in 2% agarose gels with ethidium bromide staining. The reaction mixtures without genomic templates were used as a negative control.

### The Multiplex ET-PCR Reaction

For the multiplex reactions, three sets of ET-PCR primers were combined into a single reaction mixture for multiplex analysis. The ET-PCR methodology was carried out as the following system: 25 μl reaction volume containing 0.2 μM S. p-EF, 0.2 μM S. p-R primer, 0.2 μM S. a-EF, 0.2 μM S. a-R primer, 0.2 μM E. f-EF, 0.2 μM E. f-R primer, 2 × Premix (Takara Bio, Inc., Otsu, Japan) Ex Taq TM, 1.5 μl (15 U) of BstUI endonuclease, and 1 μl each of *S. pneumoniae, S. aureus*, and *E. faecalis* DNA template. A series of genomic DNA templates (2.5 × 10^7^, 2.5 × 10^6^, 2.5 × 10^5^, 2.5 × 10^4^, 2.5 × 10^3^, 2.5 × 10^2^, 2.5 × 10^1^, and 2.5 × 10^0^ fg per microliter) of pure *S. pneumoniae* (ATCC 700674), *S. aureus* (ICDC-NP001) and *E. faecalis* (ATCC 51299) strains were applied to test the analytical sensitivity of the multiplex ET-PCR assay.

The multiplex ET-PCR mixtures were conducted using the PCR settings of pre-denaturation at 95°C for 60 s, 40 cycles of denaturation at 95°C for 5 s, and extension at 60°C for 40 s in real-time system (Rotor-Gene Q, Qiagen). Fluorescence readings were simultaneously obtained using HEX, Cy5 and FAM channels. Moreover, analysis of each dilution was determined at least four replicates, and mixtures without templates were selected as a negative control.

### Evaluation of Specificity of the ET-PCR Technique

For the ET-PCR specificity examination, the multiplex ET-PCR reaction was conducted under the conditions described above with pure genomic templates from various strains (**Table 2**). At least two replicates of each sample were independently tested.

### Practical Application of ET-PCR to *S. pneumoniae, S. aureus*, and *E. faecalis* in Blood Samples

The human blood samples were acquired from a healthy donor with the written informed consent. Our study was reviewed and approved by the ethics committee of the National Institute for Communicable Disease Control and Prevention, China CDC, according to the medical research regulations of the Ministry of Health China (Approval No. ICDC2014003).

In order to evaluate the utility of ET-PCR technique, three strains, including *S. pneumoniae* (ATCC 700674), *S. aureus* (ICDC-NPSau001), and *E. faecalis* (ATCC 51299), were used to test the sensitivity of the ET-PCR in blood samples. Firstly, three bacteria suspensions (with an optical density at 0.6), which contained *S. pneumoniae, S. aureus*, and *E. faecalis* strains, respectively, were prepared in 1 ml of sterile saline. The numbers of colony forming units (CFU) in the three suspensions were estimated using a plate-counting method according to previous report (Harris et al., 2008). In brief, serial 10-fold dilution (10^-1^–10^-8^) of three suspensions was performed, and the aliquots of 100 μl appropriate dilution (10^-6^) were plated on blood agar in triplicate. The numbers of CFU were counted after 24 h at 37°C. Then, the aliquots 100 μl of each suspension with appropriate dilutions (10^-3^, 10^-4^, 10^-5^, 10^-6^, and 10^-7^) were simultaneously added into the blood samples (700 μl), and the numbers of *S. pneumoniae* were adjusted to approximate 6500, 650, 65, 6.5, and 0.65 CFU/ml, *S. aureus* for 68000, 6800, 680, 68, and 6.8 CFU/ml, and *E. faecalis* for 71000, 7100, 710, 71, and 7.1 CFU/ml. Aliquots (100 μl) of the artificially contaminated blood samples were subjected to extract genomic DNA, and the templates were eluted in 10 μl of Qiagen elution buffer. Aliquots (2 μl) of extracted DNA were used for ET-PCR, qPCR (SYBR Green) and end-point PCR reaction. Non-contamination blood sample was chosen as negative control. This performance was independently conducted in twice.

## Results

### The ET-PCR Design

The ET-PCR design and reaction mechanism is depicted in **Figure 1**. The ET-PCR chemistry, which integrates PCR technique, restriction endonuclease cleavage and real-time fluorescence detection methodology, provides an appealing strategy for nucleic acid analysis. For the ET-PCR chemistry, the traditional forward primer (F) or reverse primer (R) was modified at 5′ end with a short sequence (Ss) that can be recognized by restriction enzyme BstUI, and the novel F or R primer was called EF or ER. The novel EF or ER is labeled with a reporter dye at 5′ end and in the middle with a quenching dye (**Figure 1A**). The quenching dye and reporter dye are very close to each other, thus this effectively prevent the emitted fluorescence of the reporter molecule. One reporter dye is assigned to one ET-PCR primer set, thus the ET-PCR chemistry achieve real-time analysis of multiple, distinct sequences in a single vessel using the novel EF or ER primer.

In ET-PCR system, the restriction enzyme BstUI, which recognize target sequence 5′-CGCG-3′ and cut sequence 5′-CG-3′ at temperatures from 55 to 65°C, is used for the ET-PCR chemistry. Importantly, the nuclease activity of BstUI does not be destroyed at high temperature, when the double-stranded DNA is denatured into single-stranded DNA. To protect the recognition site, an additional base (T) was added to the 5′ end of target recognition site (5′-CGCG-3′). The only constraint is that the targeted region must not contain this restriction site. In order to construct EF or ER primer, the sequence 5′-TCGCG-3′ can be attached to the 5′ end of any F or R primers, and the EF or ER maintains its function as a conventional PCR primer with the added advantage of simultaneous real-time analysis of the ET-PCR reaction by generating the fluorophore signal (**Figure 1B**).

### The Primer Design of ET-PCR Chemistry

For demonstrating the principle of ET-PCR technique, we chose the *ply, nuc*, and *Ef0027* genes, which were specific for *S. pneumoniae, S. aureus*, and *E. faecalis* species, as model targets. Three sets of ET-PCR primers, which only required recognition of two distinct regions on the target sequences, were designed by using PRIMER PREMIER software version 5.0, just like conventional PCR chemistry. The specificity of ET-PCR primers was determined using the National Center for Biotechnology Information Basic Local Alignment Search Tool. The details of ET-PCR primer design, sequence and location were listed in **Figure 2** and **Table 1**.

### Confirmation of ET-PCR Products

**FIGURE 3:**
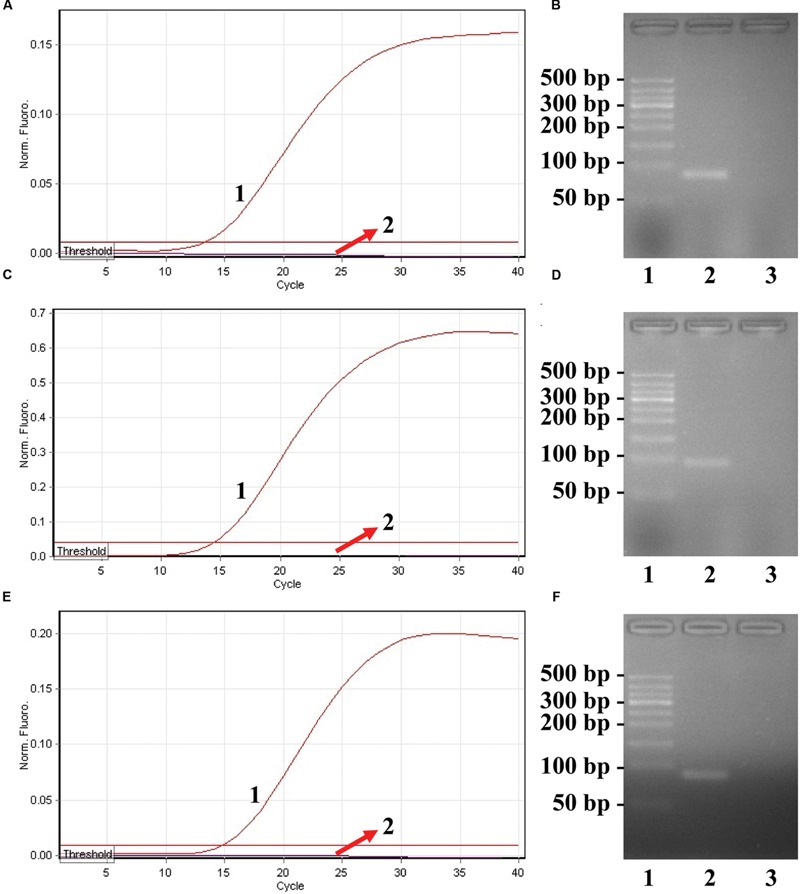
**Confirmation and detection of ET-PCR products. (A)**, **(C)**, and **(E)**, the ET-PCR was detected by means of real-time format, and the three figures were obtained from HEX (labeling S. p-EF of *ply*), Cy5 (labeling S. a-EF of *nuc*), and FAM (labeling E. f-EF of *Ef0027*) channels. Signals A1, C1, and E1 indicate *Streptococcus pneumoniae, Staphylococcus aureus*, and *Enterococcus faecalis* strains in HEX, Cy5, and FAM channels, respectively, and signals A2, C2, and E2 for negative control. **(B)**, **(D)**, and **(F)**, agarose gel electrophoresis applied to ET-PCR products; lane 1, DL 50-bp DNA markers, lane 2, positive ET-PCR products; lane 3, negative control (no DNA).

In order to confirm the correct amplification of ET-PCR, the ET-PCR reactions were conducted in the presence or absence of templates according to the standard system. Two monitor techniques, including real-time detection and agarose gel electrophoresis, were used for analyzing the ET-PCR reaction. As expected, the release of quenching was observed as a robust increase of HEX, Cy5, and FAM signals in the positive results, but not in negative control (**Figures 3A,C,E**). The ET-PCR products were electrophoresed to demonstrate the presence of the expected 89-bp (*S. pneumoniae*), 93-bp (*S. aureus*), and 80-bp (*E. faecalis*) bands (**Figures 3B,D,F**). Hence, each pair of ET-PCR primers could amplify the predicted product specifically from the genomic templates of the corresponding pathogen, which was demonstrated by real-time detection and agarose gel electrophoresis.

### Real-Time Analysis of a Single Target in an ET-PCR Reaction

**FIGURE 4:**
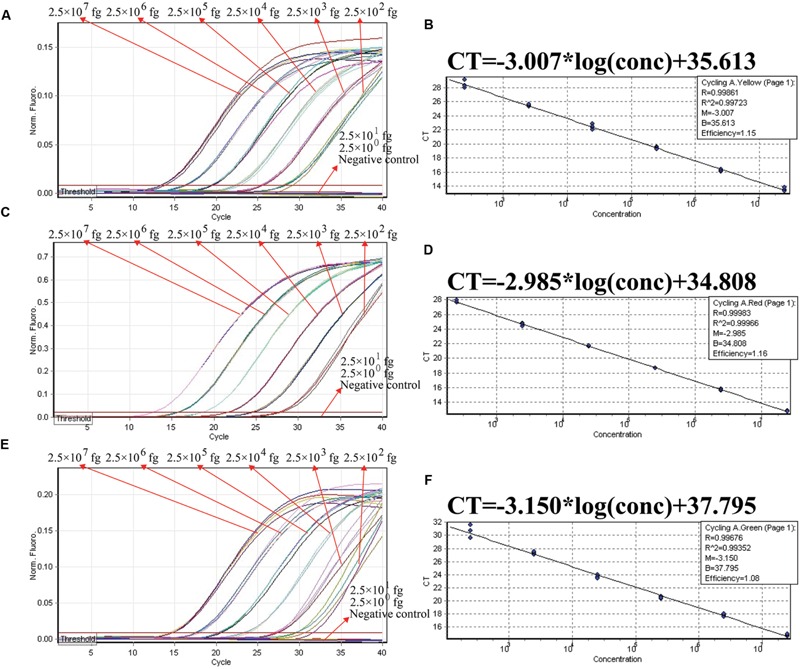
**The analytical sensitivity of ET-PCR assay for detecting a single target.** Three sets of ET-PCR primer targeting *ply, nuc*, and *Ef0027* genes were in different reactions: *S. pneumoniae* **(A)**, *S. aureus* **(C)**, and *E. faecalis* **(E)**. The DNA levels from 2.5 × 10^7^ to 2.5 × 10^2^ fg per tube provide the positive signals, 2.5 × 10^1^ and 2.5 × 10^0^ fg per reaction and negative control generate negative signal. (**B;** *S. pneumoniae*), (**D**; *S. aureus*), and (**F**; *E. faecalis*), *C*_t_ was plotted against the sample DNA concentration (fg) of the reactions.

**FIGURE 5:**
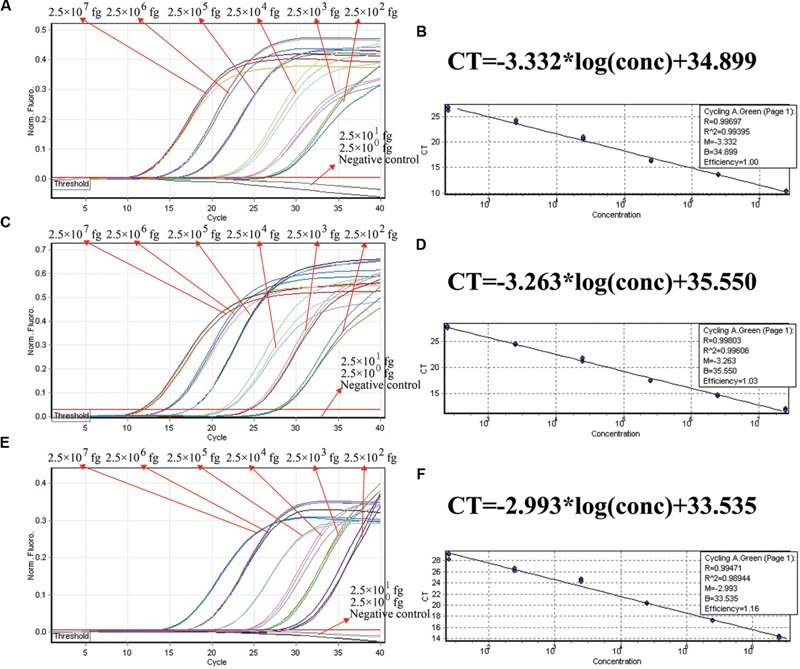
**The analytical sensitivity of conventional qPCR (SYBR Green) assay for detecting a single target.** Three sets of conventional qPCR primer targeting *ply, nuc*, and *Ef0027* genes were in different reactions: *S. pneumoniae* **(A)**, *S. aureus* **(C)**, and *E. faecalis* **(E)**. The DNA levels from 2.5 × 10^7^ to 2.5 × 10^2^ fg per tube provide the positive signals, 2.5 × 10^1^ and 2.5 × 10^0^ fg per reaction and negative control generate negative signal. (**B;** *S. pneumoniae*), (**D**; *S. aureus*), and (**F**; *E. faecalis*), *C*_t_ was plotted against the sample DNA concentration (fg) of the reactions.

**FIGURE 6:**
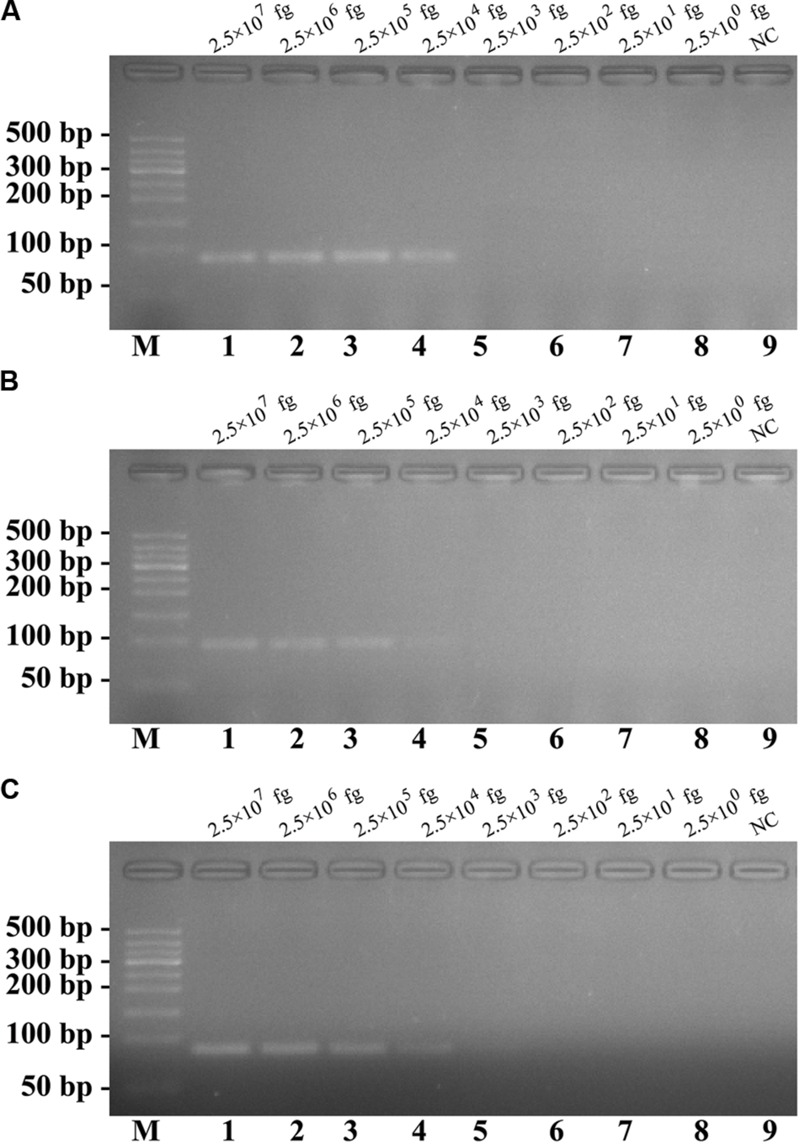
**The analytical sensitivity of conventional PCR assay for detecting a single target.** Three sets of conventional PCR primer targeting *ply, nuc*, and *Ef0027* genes were in different reactions: *S. pneumoniae* **(A)**, *S. aureus* **(B)**, and *E. faecalis* **(C)**. Agarose gel electrophoresis was applied to monitor the traditional PCR products; lane M, DL 50-bp DNA markers, lanes 1–8, the genomic DNA levels of 2.5 × 10^7^, 2.5 × 10^6^, 2.5 × 10^5^, 2.5 × 10^4^, 2.5 × 10^3^, 2.5 × 10^2^, 2.5 × 10^1^, and 2.5 × 10^0^ fg per reaction; lane 9, NC (negative control). The DNA levels from 2.5 × 10^7^ to 2.5 × 10^4^ fg per tube provide the positive signals, 2.5 × 10^3^ and 2.5 × 10^0^ fg per reaction and negative control generate negative signal.

We examined the ET-PCR analysis in a single target format by separately amplifying *ply* (*S. pneumoniae*-specific gene), *nuc* (*S. aureus*-specific gene), and *Ef0027* (*E. faecalis*-specific gene) with pure genomic templates from *S. pneumoniae* (ATCC700674), *S. aureus* (ICDC-NP001), and *E. faecalis* (ATCC51299), respectively. These fluorescent intensity-reaction cycle curves synchronized very well among four replicates containing same dilution of target DNA. The release of quenching was obtained from 2.5 × 10^7^ to 2.5 × 10^2^ fg of sample DNA, and the HEX, Cy5 and FAM signals corresponded to *S. pneumoniae, S. aureus*, and *E. faecalis* detection, respectively (**Figures 4A,C,E**). In order to quantitatively analyze the ET-PCR reactions, the plot of initial amount of sample DNA for a set of known standards (10-fold serial dilution) versus CT (threshold cycle) was a straight line (the standard curve), which was analogy to the others real-time PCR analysis (**Figures 4B,D,F**). The analytical sensitivity of ET-PCR for *S. pneumoniae, S. aureus*, and *E. faecalis* detection was 2.5 × 10^2^ fg per reaction, which was in conformity with the conventional qPCR (SYBR) analysis and 100-fold more sensitive than end-point PCR detection (**Figures 5** and **6**; **Table 3**).

**Table 3:** The analytical sensitivity for ET-PCR targeting *ply, nuc*, and *Ef0027* genes compared with that of conventional qPCR (SYBR Green) and PCR approaches.

Assays	Regions recognized	Multiplex detection	LoD for *S. pneumoniae*/S. *aureus*/*E. faecalis* (fg/reaction)	Fastest time (minutes)	LoD time (minutes)
S-ET-PCR	2	-	2.5 × 10^2^/2.5 × 10^2^/2.5 × 10^2^	21	49
M-ET-PCR	2	+	2.5 × 10^2^/2.5 × 10^2^/2.5 × 10^2^	24	55
qPCR	2	-	2.5 × 10^2^/2.5 × 10^2^/2.5 × 10^2^	20	49
PCR	2	-	2.5 × 10^4^/2.5 × 10^4^/2.5 × 10^4^	150	150

*LoD, limit of detection; S-ET-PCR, singlex ET-PCR; M-ET-PCR, multiplex ET-PCR. The LoD values are the lowest genomic DNA level that was positively amplified in triplicate. The positive results of ET-PCR and qPCR were obtained as *c*_t_ values, which converted to time for detection.*

### Real-Time Analysis of Multiplex Targets in an ET-PCR Reaction

**FIGURE 7:**
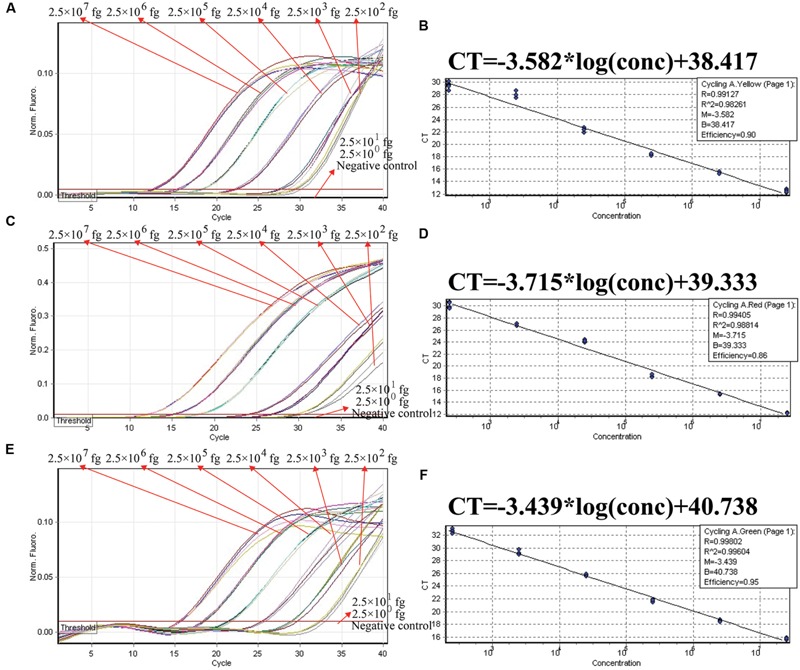
**The analytical sensitivity of ET-PCR assay for simultaneously detecting three targets.** Three sets of ET-PCR primer targeting *ply, nuc*, and *Ef0027* genes were used in the same reaction. **(A)**, **(C),** and **(E)** were simultaneously yielded from HEX (labeling EF of *ply*), Cy5 (labeling EF of *nuc*), and FAM (labeling EF of *Ef0027*), respectively. The DNA levels from 2.5 × 10^7^ to 2.5 × 10^2^ fg per tube provide the positive signals, 2.5 × 10^1^ and 2.5 × 10^0^ fg per reaction and negative control generate negative signal. (**B**; *S. pneumoniae*), (**D**; *S. aureus*), and (**F**; *E. faecalis*), *C*_t_ was plotted against the sample DNA concentration (fg) of the reactions.

In order to ensure simultaneous, reliable and correct amplification of multiple target pathogens, the multiplex ET-PCR assay was designed based on the singlex ET-PCR assays above. The three sets of primers were combined into a single reaction mixture for multiplex ET-PCR analysis, and the amount of three primer pairs was adjusted to achieve rubost multiplex ET-PCR analysis and eliminate the interaction occurred between the three primer sets. The ET-PCR amplification of each genomic DNA was unaffected by the presence of templates (2.5 × 10^7^ to 2.5 × 10^2^ fg) from the other two targets. In the multiplex ET-PCR reactions, the analytical sensitivity for *S. pneumoniae, S. aureus*, and *E. faecalis* detection also was 250 fg per reaction, which was in conformity with singlex ET-PCR detection (**Figures 7A,C,E**). Three targets, which were simultaneously added into a single reaction mixture, could be correctly identified by the multiplex ET-PCR analysis. Furthermore, three standard curves were also generated in the three-targets analysis by ET-PCR chemistry, which could be applied for quantitation of the amount of target in the unknown samples (**Figures 7B,D,F**).

### Analytical Specificity of the Multiplex ET-PCR Methodology

**FIGURE 8:**
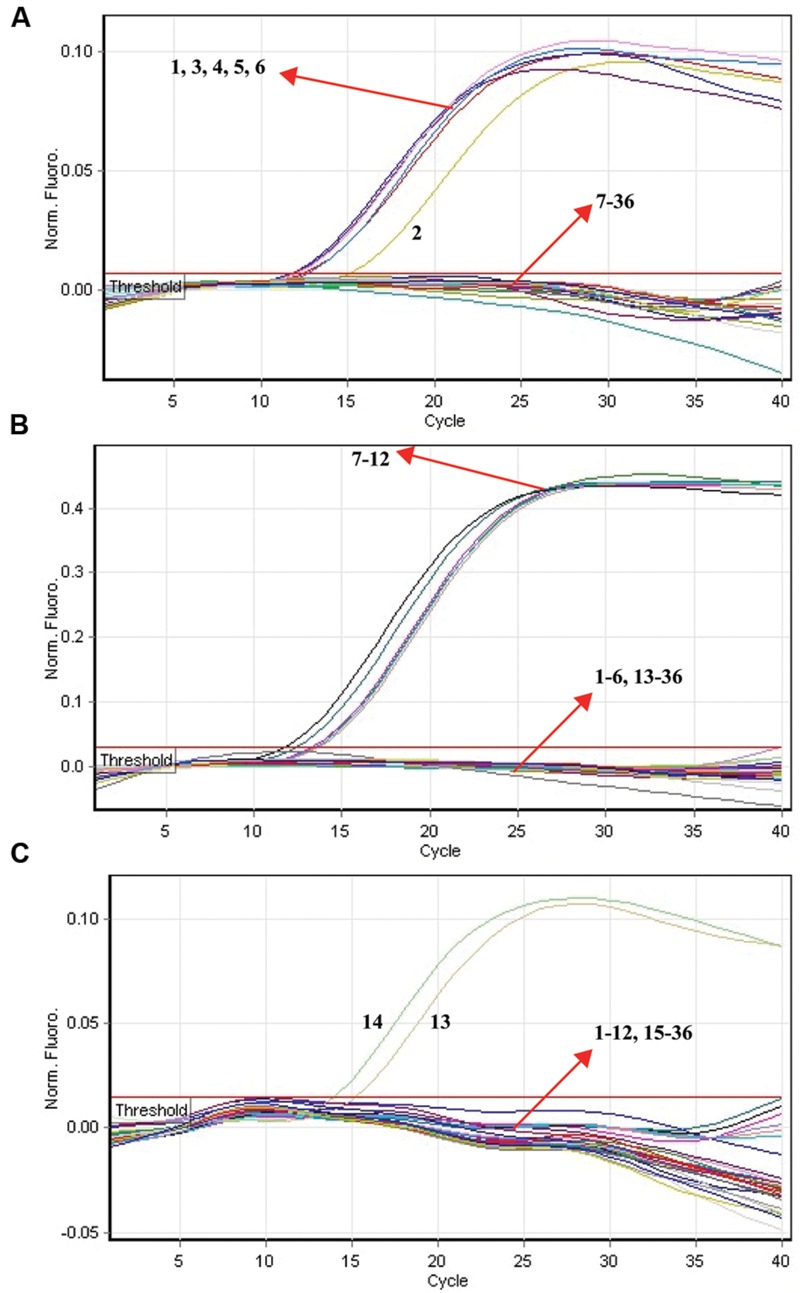
**The analytical specificity of multiplex ET-PCR detection of different strains.** The multiplex ET-PCR reactions were carried out using different genomic templates and were analyzed by means of real-time detection. **(A–C)** were simultaneously generated from HEX, Cy5, and FAM channels, respectively. Signals 1-6, strains of *S. pneumoniae* (ATCC6314), *S. pneumoniae* (ATCC49619), *S. pneumoniae* (ATCCBAA660), *S. pneumoniae* (ATCC700669), *S. pneumoniae* (ATCCBAA659), *S. pneumoniae* (ATCCBAA658); signals 7–12, *S. aureus* (ICDC-NPSau001), *S. aureus* (ICDC-NPSau002), *S. aureus* (ICDC-NPSau003), *S. aureus* (ICDC-NPSau004), *S. aureus* (ICDC-NPSau005), *S. aureus* (ICDC-NPSau006, Isolated strains); signals 13–14, *E. faecalis* (ATCC35667), and *E. faecalis* (ICDC-NPEf001). Signals 13–35, non-*S. pneumoniae*, non-*S. aureus*, non-*E. faecalis* strains of *Enteroinvasive Escherichia coli, Enteropathogenic E. coli, Enterotoxigenic E. coli, Enteroaggregative E. coli, Enteroaggregative E. coli, Enterohemorrhagic E. coli, Enterobacter cloacae, Plesiomonas shigelloides, Yersinia enterocolitica, Bntorobater sakazakii, Vibrio cholera, Shigella boydii, Shigella flexneri, Vibrio parahaemolyticus, Enterotoxigenic faecium, Listeria monocytogenes, V. vulnificus, Salmonella, Campylobacter jejuni, Bacillus cereus*, and *Pseudomonas aeruginosa*, signal 36, negative control.

The specificity of multiplex ET-PCR assay was examined in relation to data with purely genomic templates from 44-*S. pneumoniae*, 25-*S. aureus*, 22-*E. faecalis*, 21 non-*S. pneumoniae*, non-*S. aureus*, and non-*E. faecalis* strains (**Table 2**). The positive fluorescence signals were yielded only when genomic DNAs of *S. pneumoniae, S. aureus*, and *E. faecalis* strains were use as the templates for ET-PCR analysis, and the target pathogens could be correctly differentiated (**Figure 8**). In contrast, no positive signals were generated with any of the samples from non-*S. pneumoniae*, non-*S. aureus*, non-*E. faecalis* strains and negative control. These results suggested the specificity of ET-PCR assay was 100% in this report.

### Practical Application of ET-PCR to Target Pathogens in Blood Samples

In order to validate the usability of ET-PCR chemistry as a nucleic acid analysis tool, we analyzed the artificially contaminated blood samples with three target microorganisms by using ET-PCR and conventional approaches. The multiplex ET-PCR assay generated the positive fluorescence signals when the contaminated numbers of *S. pneumoniae, S. aureus*, and *E. faecalis* were more than 650 CFU/ml (~13 CFU/reaction), 680 CFU/ml (~13.6 CFU/reaction), and 710 CFU/ml (~14.2 CFU/reaction), respectively, and the three targets were simultaneously distinguished in the same sample (**Table 4**). No positive signals were obtained from negative control (non-contaminated blood samples). Comparatively, the analytical sensitivity of ET-PCR for blood samples was consistent with the conventional qPCR (SYBR) analysis and 10-fold more sensitive than that of end-point PCR approach (**Table 4**).

**Table 4:** Comparison of ET-PCR, qPCR (SYBR) and PCR assays for detection of *S. pneumonia, S. aureus*, and *E. faeclis* in artificially contaminated blood samples.

Detection methods^a^	Multiplex detection	LoD (no./reaction)		
		*S. pneumonia* detection	*S. aureus* detection	*E. faecli* detection
M-ET-PCR	+	13 CFU ~ (650 CFU/ml)	13.6 CFU ~ (680 CFU/ml)	14.2 CFU ~ (710 CFU/ml)
qPCR	-	13 CFU ~ (650 CFU/ml)	13.6 CFU ~ (680 CFU/ml)	14.2 CFU ~ (710 CFU/ml)
PCR	-	130 CFU ~ (6500 CFU/ml)	136 CFU ~ (6800 CFU/ml)	142 CFU ~ (7100 CFU/ml)

*^a^M-ET-PCR, multiplex ET-PCR.*

## Discussion

A novel ET-PCR chemistry, which merged the conventional PCR chemistry and restriction endonuclease digestion with the use of fluorescent reporter dye for monitoring the production of PCR products during each amplification cycle, was successfully devised and evaluated for nucleic acid analysis in this study. The ET-PCR technique combined the advantage of conventional PCR assay in which one pair of primers was required and real-time detection systems. The performers could use the PCR primer design software (e.g., Blast Primer, DNAMAN, Primer 5) to design a pair of the ET-PCR primer, thus the primer design for ET-PCR was very convenient and easy, just like PCR. Then, only a short sequence (Ss, 5′-TCGCG-3′) was attached to the 5′ end of PCR primer (F/R) to construct the EF/ER primer. In order to perform the ET-PCR assay, a target sequence, even as short as 40 base pairs, could be used for designing ET-PCR primers to conduct the ET-PCR test. The ET-PCR technique could continuously analyze the PCR products formation in the entire process by measuring the fluorescent signals generated from specific labeled EF/ER primer, eliminating a post-PCR analysis, thus had advantages on low contamination risk and less hand-on time.

In the ET-PCR system, EF/ER primer was modified at the 5′ end with a reporter dye and in the middle with a quenching molecule, and one reporter molecule corresponding to fluorescent channel was assigned to one target. Hence, the ET-PCR could be in singlex or multiplex analysis. The key feature of the ET-PCR chemistry was use of BstUI for specifically cutting the double-stranded recognition sequences (Ss, 5′-TCG-3′ and its Cs) during the extension phase, which separated the reporter molecule from the quenching dye leading to a fluorescent signal proportional to the amount of PCR products (**Figure 1**). Thus, the fluorescence was also measured during this phase. Importantly, this process was repeated in each ET-PCR cycle and unaffected the exponential synthesis of the PCR amplicons. By plotting cycle number versus fluorescence increment, the ET-PCR system yielded a graph that offered a more complete picture of the reaction process (**Figures 4** and **7**). Moreover, BstUI could perform the digestion of target site from 55 to 65°C, which allowed researchers to set the *T*_m_ value of ET-PCR primers with greater flexibility according to the characteristics of target nucleic acid sequences (e.g., GC content).

The ET-PCR chemistry presented here displayed advantages over existing several fluorescent systems, which were established for real-time PCR (Kubista, 2012). First, the ET-PCR technique did not require any intercalating dyes (e.g., SYBR green), obviating the disadvantages posed by intercalating molecules, for example high tendency to inhibit PCR (Monis et al., 2005). Second, an additional fluorescence probe (e.g., TaqMan probe), which was complementary to a sequence locating between the PCR primers, was not included in the ET-PCR system, simplifying the primer design and reducing the cost (Gibson et al., 1996; Wilson, 2013). Third, ET-PCR technology did not rely on the complex hairpin/stem-and-loop configuration (e.g., molecular beacons), which could lead to dimer-primers or amplification of unspecific products (Kubista, 2012). Fourth, due to only require one pair of primers, the great advantage of ET-PCR technology was for multiplexing, where complementarity could be effectively avoided between primers and probes. Hence, several target sequences were amplified in the same vessel and differentiated in parallel. Theoretically, in *n*-plex ET-PCR reaction, there were 2*n* types of primers in one vessel when *n*-plex ET-PCR were conducted for detecting *n* different target sequences, which was similar to multiplex PCR assay. The primer design of multiplex ET-PCR assay is very important, as several sets of ET-PCR primers should have similar annealing temperature to ensure the robust generation of reliable ET-PCR products. Moreover, the concentration of ET-PCR primers should be adjusted to achieve the optimal amplification of multiplex ET-PCR technique, because interaction may occur between the multiple sets of ET-PCR primers, such as primer dimers.

As a proof of concept, three pathogens (*S. pneumoniae, S. aureus*, and *E. faecalis*), which were responsible for the majority of episodes of bacteremia in patients admitted to the intensive care unit (ICU), were employed to demonstrate the mechanism and applicability of ET-PCR chemistry for nucleic acid analysis (de Smet et al., 2011). In the singlex ET-PCR reaction, the analytical sensitivity for *S. pneumoniae, S. aureus*, and *E. faecalis* was 2.5 × 10^2^ fg purified DNA per tube, which was 10-fold more sensitivity than end-point PCR assays. Then, we combined primers for three target pathogens in a multiplex ET-PCR assay, and the LoD (2.5 × 10^2^ fg DNA of each target) was in complete accordance with the single-target detection. Thus, the ET-PCR amplification of each target was unaffected by presence of templates from the other two pathogens in a single tube assay. The newly established ET-PCR approach could correctly distinguish the three pathogens in a single reaction tube with easily interpretable results. Furthermore, the strong linear correlation (*R*^2^ = 0.988–0.999) between the amount of target templates in the singlex/multiplex ET-PCR reaction and the associated *C*_t_ values over a dynamic range of DNA concentrations (2.5 × 10^7^ to 2.5 × 10^2^ fg/reaction in the pure culture) verified the quantitative capability of ET-PCR approach when detecting the target pathogens in singlex and multiplex reactions (**Figures 4** and **7**). Then, the practical application of ET-PCR approach was successfully determined by detecting the target pathogens in blood samples with high sensitivity and specificity (**Table 4**). For the ET-PCR assay specificity examination, the positive results were generated from *S. pneumoniae, S. aureus*, and *E. faecalis* stains, and no positive fluorescence signal was observed in the assay of non-*S. pneumoniae*, non-*S. aureus*, non-*E. faecalis* stains (**Figure 8**).

## Conclusion

The ET-PCR chemistry, which is an extension of conventional PCR chemistry to accommodate real-time analysis of nucleic acid sequences, has the advantages on simple design of primer, high sensitivity and specificity, less hand-on time, low contamination risk, and multiplex analysis. Therefore, these traits will motivate the researchers to explore its application in clinical diagnostics, basic research, agriculture, and many other fields.

## Author Contributions

YiW, JX, and CY conceived and designed the experiments. YiW, YaW, LZ, LL, DL, ML, SH, DJ, HL, and XC performed the experiments. YiW, YaW, LZ, LL, DL, ML, SH, DJ, HL, and XC contributed reagents/materials/analysis tools. YiW and YaW analyzed the data. YiW, JX, and CY wrote the paper.

## Conflict of Interest Statement

YiW and CY have filed for a patent from the State Intellectual Property Office of China, which covers the novel technique and sequences included in the study (Application number CN 201610399549.1). All the other authors declare that the research was conducted in the absence of any commercial or financial relationships that could be construed as a potential conflict of interest.

## References

[B1] Arya M. Shergill I. S. Williamson M. Gommersall L. Arya N. Patel H. R. (2005). Basic principles of real-time quantitative PCR. *Expert Rev. Mol. Diagn.* 5 209–219. 10.1586/14737159.5.2.209 15833050

[B2] Corless C. E. Guiver M. Borrow R. Edwards-Jones V. Fox A. Kaczmarski E. (2001). Simultaneous detection of *Neisseria meningitidis, Haemophilus influenzae*, and *Streptococcus pneumoniae* in suspected cases of meningitis and septicemia using real-time PCR. *J. Clin. Microbiol.* 39 1553–1558. 10.1128/JCM.39.4.1553-1558.2001 11283086 PMC87969

[B3] de Smet A. M. G. Kluytmans J. A. Blok H. E. Mascini E. M. Benus R. F. Bernards A. T. (2011). Selective digestive tract decontamination and selective oropharyngeal decontamination and antibiotic resistance in patients in intensive-care units: an open-label, clustered group-randomised, crossover study. *Lancet Infect. Dis.* 11 372–380. 10.1016/S1473-3099(11)70035-4 21420908

[B4] Ettenauer J. Pinar G. Tafer H. Sterflinger K. (2014). Quantification of fungal abundance on cultural heritage using real time PCR targeting the β-actin gene. *Front. Microbiol.* 5:262 10.3389/fmicb.2014.00262 PMC4035567 24904567

[B5] Gašparič M. B. Tengs T. La Paz J. L. Holst-Jensen A. Pla M. Esteve T. (2010). Comparison of nine different real-time PCR chemistries for qualitative and quantitative applications in GMO detection. *Anal. Bioanal. Chem.* 396 2023–2029. 10.1007/s00216-009-3418-0 20087729

[B6] Gibson U. E. Heid C. A. Williams P. M. (1996). A novel method for real time quantitative RT-PCR. *Genome Res.* 6 995–1001. 10.1101/gr.6.10.995 8908519

[B7] Harris K. A. Turner P. Green E. A. Hartley J. C. (2008). Duplex real-time PCR assay for detection of *Streptococcus pneumoniae* in clinical samples and determination of penicillin susceptibility. *J. Clin. Microbiol.* 46 2751–2758. 10.1128/JCM.02462-07 18562586 PMC2519471

[B8] Kubista M. (2012). I see the light! and I see it again and again! *Clin. Chem.* 58 1505–1506. 10.1373/clinchem.2012.194126 22991422

[B9] Kubista M. Andrade J. M. Bengtsson M. Forootan A. Jonák J. Lind K. (2006). The real-time polymerase chain reaction. *Mol. Aspects Med.* 27 95–125. 10.1016/j.mam.2005.12.007 16460794

[B10] Law J. W. Ab Mutalib N. S. Chan K. G. Lee L. H. (2015). Rapid methods for the detection of foodborne bacterial pathogens: principles, applications, advantages and limitations. *Front. Microbiol.* 5:770 10.3389/fmicb.2014.00770 PMC4290631 25628612

[B11] Liu D. Wang C. Swiatlo E. J. Lawrence M. L. (2005). PCR amplification of a species-specific putative transcriptional regulator gene reveals the identity of *Enterococcus faecalis*. *Res. Microbiol.* 156 944–948. 10.1016/j.resmic.2005.05.004 16024229

[B12] Monis P. T. Giglio S. Saint C. P. (2005). Comparison of SYTO9 and SYBR green i for real-time polymerase chain reaction and investigation of the effect of dye concentration on amplification and DNA melting curve analysis. *Anal. Biochem.* 340 24–34. 10.1016/j.ab.2005.01.046 15802126

[B13] Moore P. (2005). PCR: replicating success. *Nature* 435 235–238. 10.1038/435235a 15889100

[B14] Mullis K. B. Faloona F. A. (1987). [21] Specific synthesis of DNA in vitro via a polymerase-catalyzed chain reaction. *Methods Enzymol.* 155 335–350. 10.1016/0076-6879(87)55023-6 3431465

[B15] Navarro E. Serrano-Heras G. Castaño M. Solera J. (2015). Real-time PCR detection chemistry. *Clin. Chim. Acta* 439 231–250. 10.1016/j.cca.2014.10.017 25451956

[B16] Oppliger A. Masclaux F. G. Niculita-Hirzel H. (2011). Assessment of airborne microorganisms by real-time PCR: optimistic findings and research challenges. *Front. Biosci. (Schol. Ed.)* 3:445–453. 10.2741/s163 21196388

[B17] Wang Y. Wang Y. Lan R. Xu H. Ma A. Li D. (2015a). Multiple endonuclease restriction real-time loop-mediated isothermal amplification: a novel analytically rapid, sensitive, multiplex loop-mediated isothermal amplification detection technique. *J. Mol. Diagn.* 17 392–401. 10.1016/j.jmoldx.2015.03.002 26094089

[B18] Wang Y. Wang Y. Luo L. Liu D. Luo X. Xu Y. (2015b). Rapid and sensitive detection of *Shigella* spp. and *Salmonella* spp. By multiple endonuclease restriction real-time loop-mediated isothermal amplification technique. *Front. Microbiol.* 6:1400 10.3389/fmicb.2015.01400 PMC4677097 26697000

[B19] Wang Y. Wang Y. Zhang L. Liu D. Luo L. Li H. (2016). Multiplex, rapid, and sensitive isothermal detection of nucleic-acid sequence by endonuclease restriction-mediated real-time multiple cross displacement amplification. *Front. Microbiol.* 7:753 10.3389/fmicb.2016.00753 PMC4870240 27242766

[B20] Wilson N. (2013). Quantitative real-time PCR in applied microbiology [Book Review]. *Aust. J. Med. Sci.* 34:70 10.3389/fmicb.2016.00753

[B21] Yang H. Ma X. Zhang X. Wang Y. Zhang W. (2011). Development and evaluation of a loop-mediated isothermal amplification assay for the rapid detection of *Staphylococcus aureus* in food. *Eur. Food Res. Technol.* 232 769–776. 10.1007/s00217-011-1442-8

